# Sex differences and risk factors for diabetes mellitus - an international study from 193 countries

**DOI:** 10.1186/s12992-018-0437-7

**Published:** 2018-11-28

**Authors:** Devy Elling, Pamela J. Surkan, Sahba Enayati, Ziad El-Khatib

**Affiliations:** 1 Department of Public Health Sciences, Stockholm University, Stockholm, Sweden; 2 Department of Public Health Sciences, Karolinska Institutet, Stockholm, Sweden; 3 Department of International Health, Johns Hopkins Bloomberg School of Public Health, Baltimore, USA; 4 Kompetenzcenter Gesundheit, St. Stephan, Wels, Austria; 5 World Health Programme, Université du Québec en Abitibi-Témiscamingue (UQAT), Québec, Canada

**Keywords:** Diabetes mellitus, Mortality, DALY, Sex difference, Global burden of disease

## Abstract

**Background:**

Increases in overweight and obesity among youths have resulted in the diagnosis of Type 2 diabetes mellitus (T2DM) at earlier ages. The impact of lifestyle-related factors has been implicated; however, its relation to morbidity and mortality and sex differences remain unclear. We aimed to document the changes in risk factors and sex differences associated with T2DM-related morbidity and mortality during 1995–2015.

**Method:**

We used mortality rates and morbidity estimates from the Global Burden of Diseases Study 2016 using Disability-Adjusted Life Years (DALY). Multiple linear regression analyses were used to determine associations between T2DM-related mortality and related risk factors. DALYs were grouped by country income level, and were stratified by sex.

**Results:**

Increases in mortality were observed for both sexes, and females tended to have higher mortality rates per 100,000 persons. Body mass index (BMI) continued to be the leading risk factor for T2DM-related mortality, and increases in BMI were more common in low- and middle-income countries (LIC and MIC). Low physical activity was strongly associated with mortality rates, followed by dietary risks and smoking (2.4; 1.4; 0.8 per 100,000 persons, respectively). Similar patterns were observed after adjustments for income level, sex, and age. DALYs continued to show increasing trends across all income levels during 1995–2015 (high-income (HIC):16%; MIC: 36%; LIC: 12%). Stratification by sex showed similar results; males had fewer T2DM DALYs than females, though a greater increase was observed among males.

**Conclusion:**

Overall, T2DM related mortality was higher among females. Compared to in HIC, there appeared to be a considerable increase in the burden of T2DM in MIC and LIC, where BMI is the leading risk factor for T2DM-related mortality. Prevention programs should emphasize related risk factors according to the existing standard of care.

## Introduction

Diabetes mellitus is a chronic non-communicable disease (NCD). Type 1 diabetes mellitus (T1DM) accounts for 5–10% of all cases globally [1], and of all the predominant Type 2 diabetes mellitus (T2DM) cases, 75% are found in low- and middle-income countries (LIC and MIC, respectively) [2, 3]. According to the 2016 World Health Organization (WHO) Global Report on Diabetes, T2DM was among the top ten leading causes of death in 2012, accounting for 1.5 million deaths worldwide [1]. However, there is limited knowledge about risk factors for T2DM and its impact on public health globally, and specifically in LIC and MIC [4].

A significant increase in T2DM prevalence has been observed over the past decades [5], with the WHO’s latest estimate being 9% in individuals above 18 years [1]. Formerly T2DM was more common among the older segment of the population in high-income countries (HIC) [6]. However, it is now becoming more prevalent in younger age groups due to the global increase in overweight and obesity, resulting in a greater burden of the disease at an earlier age [1, 7, 8].

This increased burden of T2DM may be due to lifestyle changes [9], resulting in high body-mass index (BMI), poor dietary habits and low physical activity. Moreover, smoking has been associated with increased risk of T2DM-related morbidity and mortality [3, 10]. The relationship between T2DM and high BMI depends on body fat distribution [11], though research on the effects of excess weight and life expectancy is limited [12]. In regard to factors contributing to high BMI, the importance of healthy dietary habits, such as a diet high in fruits and vegetables and low consumption of saturated fats, has been associated with reduced risk of T2DM [13]. Other foods, for instance, dairy products and different protein sources on T2DM risk, are yet to be explored [14]. Previous studies have suggested the importance of physical activity in improving glucose metabolism and controlling excess weight to lower the risk of T2DM [15, 16]. In addition, research has indicated an association between T2DM and smoking; however, the effects of smoking may be altered in the presence of other risk factors such as high BMI [16, 17].

Across the globe, females have a higher risk of T2DM compared to males due to their higher body fat composition [18, 19]. Interestingly, insulin resistance has been closely related to fat distribution around the abdominal area that is commonly found in males, contradicting earlier findings [20]. Thus, sex differences may be attributable to social and cultural factors [21], in addition to biological differences [22].

Due to recent lifestyle shifts, NCDs related-morbidity and mortality have increased [9]. To our knowledge, the impacts of related risk factors on mortality rates and sex differences in T2DM-related morbidity and mortality have not been clearly explored across different types of global economies. Thus, in the present study we aimed to observe global changes in risk factors and sex differences associated with T2DM-related morbidity and mortality between 1995 and 2015.

## Methods

For this study we used the estimates from the Global Burden of Diseases Study 2016 (GBD 2016) for T2DM-related mortality rates and morbidity using the Disability-Adjusted Life Years (DALYs) [23]. We defined diabetes cases, including both T1DM and T2DM, based on the GBD 2016 definition of diabetes. More comprehensive modeling of diabetes-related mortality in the GBD 2016 has been described [24]. We present 2015 estimates of T2DM, including associations of DM-related mortality with related risk factors, including high BMI, dietary risks, low physical activity, and smoking. Dietary risks included diet high in processed meat, red meat, sodium, sugar-sweetened beverages and trans fatty acids [25]. In addition, diet low in calcium, fiber, fruits, milk, nuts and seeds, polyunsaturated fatty acids, seafood with omega-3 fatty acids, and vegetables were also classified as dietary risks [25]. More detailed information on each of the risk factor can be found on the Institute for Health Metrics and Evaluation (IHME) database [25]. Data on only individuals aged ≥15 years were available when taking the abovementioned risk factors into account. All countries were divided into three income levels based on their Gross National Income (GNI) per capita, according to the World Bank’s definition in 2015. Countries categorized as LIC had GNI per capita of ≤$1205, whereas MIC and HIC had GNI per capita of $1026–12,475 and ≥ $12,476, respectively [26]. Morbidity using DALYs for each country income level (HIC, MIC, LIC) was calculated by summing DALYs from respective countries and dividing it by their populations. The total population in each country and the proportion of males to females were extracted from the United Nations (UN) population statistics for each year.

### Statistical analyses

Data extracted from GBD 2016 were merged by location, sex, and age. Due to the non-normally distribution of the data on aggregate level, we decided to apply non-parametric statistics for in our method of analysis. Assessment of sex differences using median rate of diabetes-related mortality and risk associated with T2DM was conducted using the Wilcoxon rank-sum test. Risk factors were tested for multicollinearity using the variance inflation factor (VIF), where a VIF > 10 was considered highly collinear. Risk factors were continuous variables, and income level, sex and age were categorical variables. Thus, multiple linear regression analysis was conducted to determine the association between T2DM-related mortality and risk factors. Adjustments for country income level, sex and age were computed, and 95% confidence intervals (95% CI) were calculated. We considered alpha-level of *p* < 0.05 significant. The prevalence of T2DM-related mortality using DALYs grouped by country income level and stratification by sex was estimated. Analyses were computed using Stata statistical software v.12 (StataCorp. 2011. *Stata Statistical Software*: Release 12. College Station, TX: StataCorp LP).

## Results

Among 193 countries from 1995 to 2015, an increase in T2DM-related mortality was observed for both sexes; females tended to have higher mortality rates per 100,000 persons compared with their male peers across country income levels (Table 1).

**Table 1:** Global diabetes mellitus-related mortality per 100,000 persons per income level (high-, middle-, and low-income countries) in 1995, 2005, and 2015 among individuals aged ≥15 years by sex

Mortality rate per 100,000 persons	1995		2005		2015	
	M	F	M	F	M	F
Globally	12.9	15.9	16.2	19.1	20.1	21.1
High-income countries	15.0	18.9	18.6	21.2	18.7	19.6
Middle-income countries	13.4	18.0	18.8	21.5	23.5	25.8
Low-income countries	9.8	10.0	10.3	10.9	11.9	12.7

*M* Males, *F* Females

A constant increase in high BMI was observed over time (10.9 to 14.6 per 100,000 persons) (Table 2), and BMI was the leading risk factor for T2DM-related mortality globally in 2015. Similar increases in BMI were observed in MIC and LIC; however, HIC showed stable rates of high BMI during 1995–2005 and a decrease in rates during 2005–2015 (10.4 to 9.3 per 100,000 persons). Over the same time period dietary risks increased in MIC and LIC, and mortality rates decreased in HIC (8.6 to 7.7 per 100,000 persons). Globally, dietary risks remained stable over time (13.4 to 13.3 per 100,000 persons). Physical inactivity did not show any noticeable change in HIC (3.6 to 3.4 per 100,000 persons). The rates of low physical activity increased in MIC (4.8 to 5.7 per 100,000 persons) and LIC (2.8 to 3.4 per 100,000 persons). In MIC and LIC, the relation between smoking and T2DM-related mortality remained stable over time (Table 2).

**Table 2:** Risk factors associated with diabetes-related mortality rates per 100,000 persons globally and in high-, middle-, and low-income countries among individuals aged ≥15 years

Characteristics of risk factor per 100,000 persons	1995	2005	2015
Globally			
High BMI	10.9	12.8	14.6
Dietary risks	13.4	13.9	13.3
Low physical activity	4.4	4.5	4.5
Smoking	0.8	0.8	0.6
High-income countries			
High BMI	10.3	10.4	9.3
Dietary risks	8.6	8.4	7.7
Low physical activity	3.6	3.8	3.4
Smoking	0.6	0.5	0.4
Middle-income countries			
High BMI	14.5	18.0	19.1
Dietary risks	14.8	15.4	15.8
Low physical activity	4.8	5.6	5.7
Smoking	0.7	0.8	0.7
Low-income countries			
High BMI	5.9	7.7	9.4
Dietary risks	9.7	11.6	11.3
Low physical activity	2.8	3.1	3.4
Smoking	0.4	0.4	0.4

Males had consistently lower mortality rates than their female peers. However, male smokers had higher T2DM-related mortality across income levels. High BMI continued to be associated with increased mortality rates across income levels, though it should be noted that the trend reversed in HIC between 2005 and 2015 (M: 11.3 to 10.8; F: 10.0 to 8.1 per 100,000 persons). Overall, physically inactive males in LIC had higher mortality compared to females at the same income levels, as well as inactive males in both MIC and HIC. DM-related mortality globally and across income levels remained unchanged in terms of smoking during the study period. With the exception of high BMI in MIC in 1995 and 2005, dietary risks in HIC in 2015, and smoking across income levels, no other risk factors showed statistical differences between sexes (Table 3).

**Table 3:** Risk factors associated with diabetes-related mortality rates per 100,000 persons by country income level (high-, middle-, and low-income) among individuals aged ≥15 years by sex

Rate of risk factor per 100,000 persons	1995				2005				2015			
	M	F	Ratio (M:F)	*p*-value	M	F	Ratio (M:F)	*p*-value	M	F	Ratio (M:F)	*p*-value
Globally												
High BMI	10.0	13.6	0.7	0.77	11.8	15.0	0.8	0.77	13.9	15.3	0.9	0.77
Dietary risks	13.0	13.4	1.0	1.00	14.2	14.2	1.0	0.77	15.4	13.1	1.2	0.56
Low physical activity	4.7	4.4	1.1	0.77	5.2	4.5	1.1	0.77	5.8	4.4	1.3	0.56
Smoking	3.5	0.3	13.0	0.1	3.5	0.2	15.5	< 0.01	3.4	0.2	19.4	< 0.01
High-income countries												
High BMI	9.6	10.7	0.9	0.80	11.3	10.0	1.1	0.40	10.8	8.1	1.3	0.15
Dietary risks	8.8	8.2	1.1	0.39	9.9	7.6	1.3	0.07	8.9	6.4	1.4	< 0.01
Low physical activity	3.5	3.7	0.9	1.00	3.9	3.7	1.0	0.41	3.4	3.3	1.1	0.20
Smoking	2.1	0.2	8.8	< 0.01	1.9	0.2	10.4	< 0.01	1.6	0.2	10.3	< 0.01
Middle-income countries												
High BMI	12.3	18.0	0.7	< 0.01	14.3	21.5	0.7	0.01	16.4	20.0	0.8	0.07
Dietary risks	14.4	16.0	0.9	0.67	15.1	16.3	0.9	0.72	15.9	15.7	1.0	0.69
Low physical activity	4.8	4.9	1.0	0.96	5.8	5.4	1.1	0.90	5.9	5.61	1.1	0.45
Smoking	3.0	0.2	15.8	< 0.01	3.3	0.2	15.2	< 0.01	3.3	0.2	19.1	< 0.01
Low-income countries												
High BMI	4.8	7.5	0.6	0.28	6.4	10.0	0.7	0.08	6.9	10.7	0.6	0.08
Dietary risks	9.7	11.4	0.9	0.58	10.7	12.4	0.9	0.67	11.1	12.2	0.9	0.61
Low physical activity	3.1	2.7	1.2	0.18	3.5	2.9	1.2	0.25	4.0	3.1	1.3	0.24
Smoking	1.3	0.1	15.1	< 0.01	1.4	0.1	20.1	< 0.01	1.6	0.1	21.1	< 0.01

*M* Males, *F* Females

We observed associations between dietary risks, low physical activity, and smoking with T2DM-related mortality. The highest increase in mortality rate was among individuals with low physical activity (2.4 per 100,000 persons), followed by those with dietary risks and among smokers (1.4 and 0.8 per 100,000 persons, respectively) prior to adjustment for country income level, sex and age. Similar associations between risk factors and T2DM-related mortality were found after adjustment for income level, sex and age. Although the adjusted association between low physical activity and T2DM-related mortality was attenuated compared to the crude model, the results still showed an increased mortality rate per 100,000 persons. MIC and LIC had higher T2DM-related mortality (13.8 and 20.4 per 100,000 persons, respectively) compared to HIC. Females across income levels had lower mortality rates compared to their male counterparts; however, this difference was not statistically significant. As expected, the age groups 50–69 years and ≥ 70 years had higher mortality than 15–49 year olds. Interestingly, the difference between the 15–49 year and 50–69 year age groups was not statistically significant (Table 4).

**Table 4:** Risks associated with diabetes mellitus-related mortality per 100,000 persons in 2015 among individuals aged ≥15 years

	Crude model	Adjusted model^a^
Risk factors		
Dietary risks	1.4 (1.1–1.6)	1.4 (1.1–1.6)
Low physical activity	2.4 (2.0–2.7)	2.0 (1.6–2.4)
Smoking	0.8 (0.4–1.2)	0.8 (0.4–1.3)
Country income level		
High-income		ref
Middle-income		13.8 (3.6–23.9)
Low-income		20.4 (6.8–34.0)
Sex		
Male		ref
Female		−1.2 (−10.5–8.1)
Age group		
15–49 years		ref
50–69 years		0.9 (−10.2–12.1)
≥ 70 years		57.7 (44.9–70.6)

^a^Adjusted for country income level, sex and age

A significant decrease in DALYs, by approximately 45%, was observed globally and across income levels from 1995 to 2005. As expected, DALYs continued to increase (HIC: 907.1 to 1052.3 years; MIC: 898.6 to 1215.8 years; LIC: 825.4 to 926.9 years) during the study period (Table 5).

**Table 5:** Global diabetes mellitus-related Disability-Adjusted Life Years (DALYs) per 100,000 years per income level in 1995, 2005, and 2015

	1995	2005	2015
Globally	896.5	522.2	1167.4
High-income countries	907.1	517.4	1052.3
Middle-income countries	898.6	531.1	1215.8
Low-income countries	825.4	421.3	926.9

T2DM burden clearly increased in both sexes over time and across income level. Globally, males showed greater increases in DALYs compared to females (M: 880.4 to 1210.7 years vs. F: 918.5 to 1123.4 years). A considerable increase of DALYs in MIC (M: 868.9 to 1243.7 years, F: 934.3 to 1187.7 years) was detected during 1995–2015. Compared to LIC and MIC, the male population in HIC did not show noticeable increase in DALYs (931.5 to 1160.2 years) compared to females (891.7 to 943.4 years). In LIC, both sexes showed trends similar to HIC and MIC (M: 844.5 to 966.6 years; F: 807.2 to 888.4 years) (Table 6).

**Table 6:** Global diabetes mellitus-related Disability-Adjusted Life Years (DALYs) per 100,000 years per income level in 1995, 2005, and 2015 by sex

	1995		2005		2015	
	M	F	M	F	M	F
Globally	880.4	918.5	1043.4	1044.0	1210.7	1123.4
High-income countries	931.5	891.7	1054.5	908.2	1160.2	943.4
Middle-income countries	868.9	934.3	1054.6	1096.1	1243.7	1187.7
Low-income countries	844.5	807.2	858.8	824.8	966.6	888.4

*M* Males*, F* Females

## Discussion

Overall, T2DM-related mortality increased globally and across all income levels, even after stratification by sex. Additionally, differences between sexes were found across all risk factors associated with T2DM-related mortality; females had higher mortality compared to males. Previous literature has highlighted gender differences in developing T2DM mainly due to biological factors [18, 22]. However, it has been suggested that global variations may be due to environmental factors, such as culture and other influences associated with socioeconomic status [18, 19, 21]. In contrast to our findings, other studies have found higher risk of T2DM-related mortality among males [21, 22]. Biological differences is a fundamental component in the development of T2DM [22]. Social and cultural issues, such as better T2DM management and help-seeking behavior are more common among females. This results in better long-term outcomes in females compared to males [21].

**Fig. 1:**
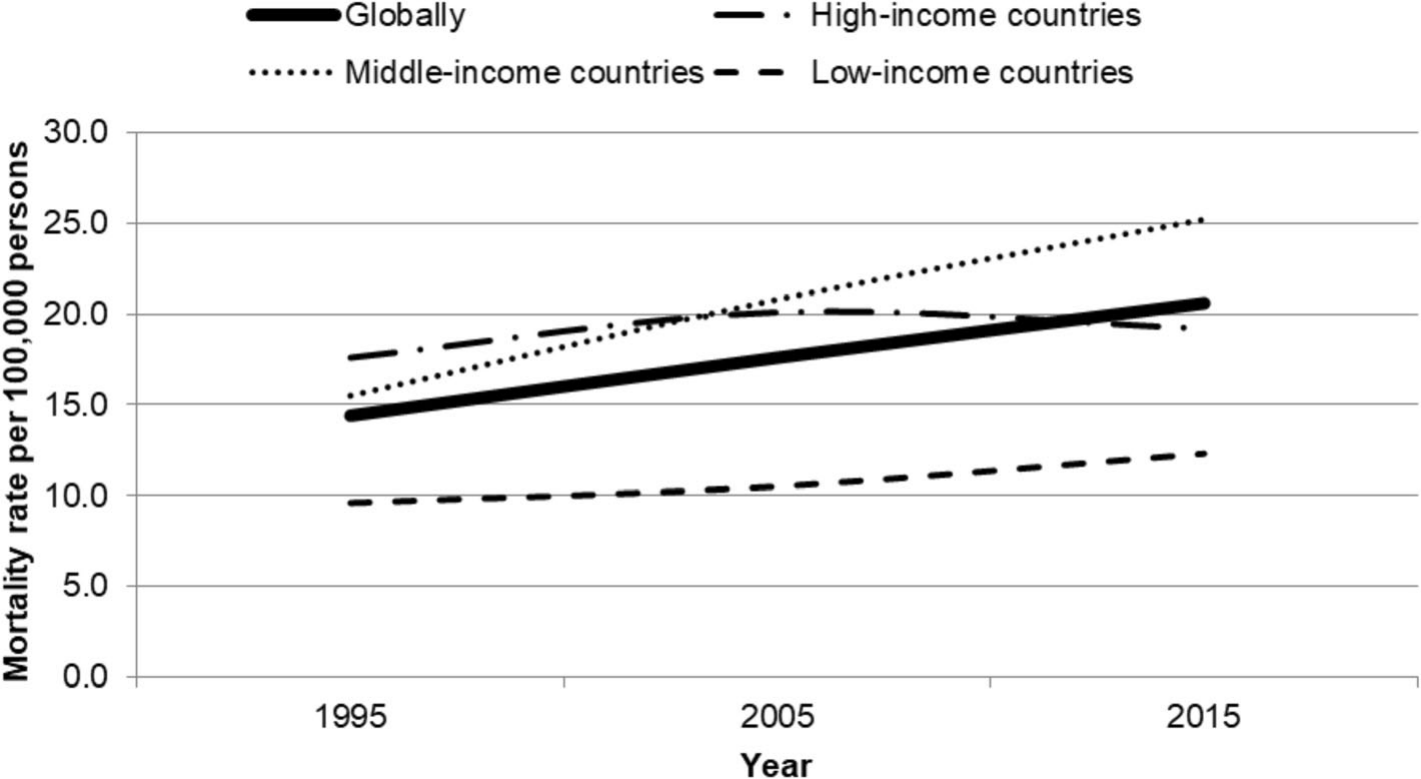
Global mortality rate per 100,000 persons by income level (high-, middle- and low-income countries) during 1995–2015 among individuals aged ≥15 years. Globally, Middle-income countries, High-income countries, Low-income countries

In HIC, fluctuation was observed in the mortality and morbidity rates of diabetes cases, likely to be T2DM, during the study period, as reflected in a decrease in rates between 2005 and 2015. This fluctuation (Fig. 1) may indicate a change in DM management, for instance through behavioral changes, leading to lower mortality [27]. When investigating sex differences, overlapping risk factors, such as high blood pressure and fat distribution, may be important indicators determining the risk of developing T2DM [19, 22]. A recent study suggested sex-specific risk factors could exacerbate complications rising from T2DM [28].

In our analysis of the association of T2DM-related mortality and lifestyle-related risk factors, high BMI was excluded due to data quality limitations and high collinearity with dietary risk factors, low physical activity, and smoking. High BMI was excluded because it is often a product of poor dietary habits and physical inactivity [29], and its importance to several chronic NCDs, including T2DM, has been highlighted previously [29, 30].

The segment of the population aged ≥ 70 years had higher mortality rates than the age group between 15 and 49 years. This is possibly due to an accumulation of risk factors and other competing health complications, for example, cardiovascular events, in the elderly population. The presence of cardiovascular events may worsen T2DM symptoms and result in a higher risk of T2DM-related complications [28]. We adjusted for country income level, sex, and age; however, it may have been beneficial to further adjust for other risk factors such as high blood pressure [30, 31]. Since this study is based on global aggregate data, we were unable to control for these variables.

While we were able to estimate T2DM-related morbidity and mortality in the present study, it is important to interpret the results with caution, as individuals suffering from DM often have comorbidities related to mental health, gastrointestinal, and musculoskeletal health [32, 33]. It is possible that the data on underlying causes of death may have been limited in many countries, resulting in an underestimation of T2DM-related mortality [34]. For instance, T2DM complications, such as cardiovascular events, may be reported as a primary cause of death [35]. In resource-constrained settings, particularly LIC, high-quality data on secondary causes of death may be limited.

Several studies have investigated disease burden due to DM, its association with other NCDs, and its impacts on the society. This present study is possibly the first study on the global level focusing on T2DM-related mortality and its relation to several major risk factors, as well as trends in DALYs over time.

High BMI was used as a T2DM indicator; however, a recent study has suggested waist circumference may be a stronger predictor of overall mortality [36]. Fat distribution in different parts of the body has been discussed as reflecting different metabolic profiles, and anthropometric data, such as BMI, may not be an adequate indicator [37]. Furthermore, high BMI was pre-defined by the GBD, i.e., a cut-off of 23 kg/m^2^ [38], and thus, the rates that we reported associated with high BMI might not reflect the true rates of overweight and obesity in the population.

We were unable to differentiate diabetes diagnoses by T1DM or T2DM, but restricted the age to ≥15 years as a means to better hone in on T2DM in the dataset. This can be considered a study limitation, as only this age group was included in the analysis. According to the definition used in this study (based on findings from previous literature, i.e., that T2DM is most common among adults) and due to the lower likelihood of T1DM in this group, the majority of our cases were likely to be T2DM. Hence, there may have been some misclassification.

GBD 2016 is a collection of several datasets, and we modeled rates to estimate certain risk factors of mortality. There is also the risk of discrepancies in data quality in different parts of the world, for instance, HIC may have better and more comprehensive data compared to MIC and LIC. GBD estimates are reviewed annually in accordance to several experts. A more thorough explanation regarding the annual review, and the process of the estimation within the GBD study, is explained elsewhere [24].

Inclusion of alcohol as a predictor of DM-related morbidity and mortality may be of interest, and would have added value to the study as alcohol may interact with other risk factors, such as dietary patterns [39, 40]. A systematic review and meta-analysis suggested effects of alcohol (beer, wine or spirits) on T2DM risk [39]. However, it was excluded from our analysis as the GBD defined alcohol consumption broadly, as any type of alcohol use, providing no information on duration, frequency or amount consumed.

The Prospective Urban Rural Epidemiology (PURE) study found an association between high carbohydrate intake and high BMI in 18 countries [41]. The PURE study found high carbohydrate intake (> 60% of energy) was consumed mainly in LIC and MIC, which was considered as the main cause of high BMI [41]. Our study provided an overview of T2DM using publicly available data from the GBD study. Lastly, a recent study by Aguirre et al. (2018) recommended the usage of waist-to-height ratio as an anthropometric measurement instead of BMI [42], which would be worth exploring in further studies.

This present study did not aim to explore the association between T2DM-related mortality and specific dietary risk factors. Different dietary habits may have altered the associations found in this study [30], and thus, more specific dietary risks, for example, the association between a diet low in dairy products and T2DM-related mortality should be explored further in future studies.

In summary, we observed an increase in T2DM-related morbidity and mortality over the last 20 years in the data gathered and analyzed based on GBD 2016. Life expectancy has increased in LIC and MIC, due to the reduction in communicable diseases, which has in turn increased the risk to acquire NCDs. This has increased awareness of T2DM in research-constrained settings and thus, prevention programs, e.g. to facilitate early diagnosis are being promoted [43].

## Conclusion

In the present study, we found considerable increase in the burden of T2DM-related in MIC and LIC compared to HIC. Additionally, females had a higher mortality rate, with BMI as the leading risk factor. Future studies should seek to understand the relation between the cumulative effects of risk factors on T2DM-related morbidity and mortality.
